# Design and Implementation of NK Cell-Based Immunotherapy to Overcome the Solid Tumor Microenvironment

**DOI:** 10.3390/cancers12123871

**Published:** 2020-12-21

**Authors:** Ishwar Navin, Michael T. Lam, Robin Parihar

**Affiliations:** 1 Department of Immunology and Microbiology, Baylor College of Medicine, Houston, TX 77030, USA; ishwar.navin@bcm.edu; 2 Center for Cell and Gene Therapy, Texas Children’s Hospital, Houston Methodist Hospital, Baylor College of Medicine, Houston, TX 77030, USA; 3 Medical Scientist Training Program and Translational Biology and Molecular Medicine Program, Baylor College of Medicine, Houston, TX 77030, USA; ml18@bcm.edu; 4 Section of Hematology/Oncology, Department of Pediatrics, Texas Children’s Hospital, Houston, TX 77030, USA

**Keywords:** NK cell, solid tumor, tumor microenvironment (TME), immunotherapy, cell therapy, chimeric antigen receptor (CAR)

## Abstract

**Simple Summary:**

Cell therapy over the last few years has revolutionized the treatment of blood cancers. However, efficacy against cancers of solid organs remains limited. These cancers pose a major challenge to the success of cell therapy by generating a suppressive environment that inhibits immunity. We review current efforts to improve the potency of natural killer (NK) cell therapy in the tumor microenvironment. The successful application of these approaches will lead to more effective treatment options and improve clinical outcomes for patients with solid cancers.

**Abstract:**

Natural killer (NK) cells are innate immune effectors capable of broad cytotoxicity via germline-encoded receptors and can have conferred cytotoxic potential via the addition of chimeric antigen receptors. Combined with their reduced risk of graft-versus-host disease (GvHD) and cytokine release syndrome (CRS), NK cells are an attractive therapeutic platform. While significant progress has been made in treating hematological malignancies, challenges remain in using NK cell-based therapy to combat solid tumors due to their immunosuppressive tumor microenvironments (TMEs). The development of novel strategies enabling NK cells to resist the deleterious effects of the TME is critical to their therapeutic success against solid tumors. In this review, we discuss strategies that apply various genetic and non-genetic engineering approaches to enhance receptor-mediated NK cell cytotoxicity, improve NK cell resistance to TME effects, and enhance persistence in the TME. The successful design and application of these strategies will ultimately lead to more efficacious NK cell therapies to treat patients with solid tumors. This review outlines the mechanisms by which TME components suppress the anti-tumor activity of endogenous and adoptively transferred NK cells while also describing various approaches whose implementation in NK cells may lead to a more robust therapeutic platform against solid tumors.

## 1. Introduction

Natural killer (NK) cells are innate immune effectors that engage in cellular cytotoxicity against virally infected or stressed cells. In contrast to T cells, which recognize targets via T cell receptors in an MHC-restricted manner, NK cells recognize target cells via a combination of activating and inhibitory signals arising from a broad range of germline-encoded cell surface receptors, including activating and inhibitory killer cell Ig-like receptors (KIRs). NK cells can thus interact with tumor targets without the need for prior antigen sensitization. Approaches to utilize the unique activity of NK cells for anti-cancer therapy have included administration of monoclonal antibodies to stimulate NK antibody-dependent cellular cytotoxicity (ADCC), utilizing MHC-KIR mismatch to enhance graft vs. leukemia effects post-hematopoietic stem cell transplant (HSCT), and most recently, ex vivo genetic modifications of NK cells to express chimeric antigen receptors (CARs) that target tumor-associated antigens (CAR-NK cells). Indeed, a recent trial of CAR-NK cells targeting the CD19 antigen in patients with acute leukemias reported impressive anti-tumor responses with no observed graft-vs-host disease (GVHD) or cytokine release syndrome (CRS), toxicities typical of other CAR-expressing cytotoxic effectors such as T cells [1,2,3]. However, treatment of solid tumors utilizing these NK cell-based approaches remains a challenge given the presence of a highly immunosuppressive solid tumor microenvironment (TME) that impairs NK functions. If NK cell-based approaches are to be effective in treating patients with solid tumors, strategies to overcome the TME must be employed. This review focuses on the various strategies that have been used to overcome the solid TME in the setting of NK cell therapies in hopes of improving efficacy in solid tumors. We review how TME components dysregulate anti-tumor functions of both endogenous and adoptively transferred NK cells, report approaches to overcome the TME using CAR-NK cells, switch receptors, dominant negative receptors, enhanced ADCC, gene ablation, and checkpoint blockade that may enhance NK cell functions, and discuss the future of TME targeting in the context of NK cell therapeutics. We hope that the compilation and discussion of this data will not only enhance our understanding of the recent biologic and technological advances in NK cells that help modulate their functions within the TME, but also pave the way for creation of more effective NK cell-based therapeutics with greater anti-tumor activity within patients with solid tumors.

## 2. The Tumor Microenvironment Dysregulates NK Cell Functions

**Figure 1:**
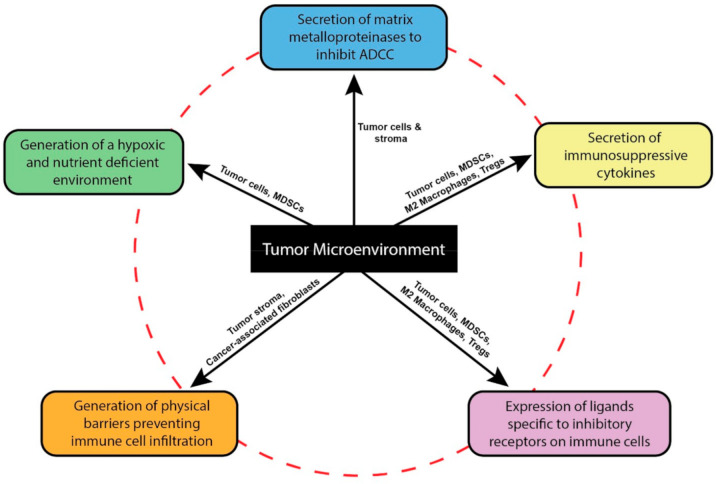
Features of the tumor microenvironment. Schematic depicting mechanisms by which components of the solid TME inhibit NK cell effector functions. Inhibitory immune cells of the TME like MDSCs, Tregs and M2 macrophages secrete multiple inhibitory cytokines (yellow) and express ligands towards checkpoint receptors on NK cells (purple) to suppress their cytotoxicity. They aid tumor in generating a hostile surrounding by depleting the TME of essential amino acids while also promoting hypoxia (green). Tumor and stromal cells of the TME secrete matrix metalloproteinases that directly inhibit antibody-dependent cellular cytotoxicity of NK cells by cleaving NK Fcγ receptors thus rendering them ineffective (blue). Additionally, stromal cells, along with cancer-associated fibroblasts generate physical barriers that inhibits the infiltration capacity of tumor infiltrating NK cells (orange).

The tumor microenvironment poses a unique chemical and physical barrier to NK cells. It comprises soluble factors such as inhibitory cytokines and metabolic products that deprive NK cells of their activity, extracellular vesicles that shuttle cytokines/chemokines, metabolic factors, and inhibitory ligands within the TME, and hypoxia rich zones affecting the metabolic fitness of NK cells. It also contains a rich cellular component consisting of tumor cells, stromal cells like cancer-associated fibroblasts, and immunosuppressive immune cells sometimes referred to as the tumor immune microenvironment (TiME) (Figure 1). TME-secreted factors like matrix metalloproteinase (MMP)-7 cleave Fas and Fas-L, preventing their interactions, as well as cleave Fcγ receptor ectodomains on NK cells [4,5]. Cancer-associated fibroblasts remodel matrix proteins and create physical barriers that inhibit effector immune cell infiltration [6,7]. Inhibitory cells of the TiME include myeloid-derived suppressor cells (MDSCs), M2 tumor-associated macrophages (M2-TAMs), T regulatory cells (Tregs), cancer associated dendritic cells, and B-regulatory cells. MDSCs consist of a heterogenous population of cells resembling immature neutrophils and monocytes [8,9,10]. These cells are able to inhibit T, NK, and dendritic cells through the expression of inhibitory ligands such as PD-L1, the release of inhibitory cytokines such as transforming growth factor (TGF)-β and IL-10, as well as expression of nutrient-depleting enzymes like Arginase-1 and IDO [11,12]. MDSCs are relatively enriched and expanded in certain cancers including liver and skin, particularly in metastatic disease settings [8,10,13,14]. Macrophages of the TiME also inhibit NK cells via similar mechanisms including release of IL-10 and TGF-β [15,16]. Additionally, inhibitory tumor-associated macrophages (M2-TAMs), utilizing similar mechanisms to MDSCs, can promote cancer growth and support metastasis [17]. T regulatory cells (CD4+/CD25^hi^/Foxp3+) also regulate the immune response in solid tumors by suppressing dendritic and NK cell mediated functions such as interferon (IFN)-γ release and expression of activating KIR, and by limiting anti-tumor efficacy through TGF-β signaling [18,19]. Serving at the interface between the adaptive and innate immune systems, tumor-infiltrating dendritic cells can promote tumor growth as they express low levels of co-stimulatory signals and high expression of regulatory molecules, leading to immunosuppression and blocking of adaptive immune responses. In addition, the protective function of dendritic cells in stimulating the anti-tumor response is blunted by tumor factors that inhibit dendritic cell maturation and function such as PD-1 expression and release of IL-10, TGF-β1, arginase-1, and IDO [20,21,22]. Lastly, and less well understood, regulatory B cells exist in many subtypes and are broadly defined by their surface marker expression. Overall, they can inhibit all critical cells of the immune response against tumors primarily through IL-10 and TGF-β expression [23]. Taken together, the TME creates an inhibitory landscape that hampers NK cell anti-tumor activity. Solid tumors offer special challenges to NK cell-based therapies secondary to this unique architecture of the TME that need to be overcome to enhance anti-tumor efficacy.

## 3. Strategies to Overcome the Tumor Microenvironment

### 3.1. CAR-NK Cell Therapy

The limited success of adoptively transferred NK cells against solid tumors can be at least partly attributed to the TME, which employs multiple mechanisms to mediate immunosuppression in regions surrounding the tumor [24,25]. Additionally, solid tumors are capable of down-regulating ligands for endogenous activating receptors on NK cells that further impedes their anti-tumor activity [26]. To help override the inhibitory signals delivered to the NK cell by the TME, and thus skew the balance of signals towards NK activation, NK cells have been genetically modified to express CARs that, depending on their design, can provide potent activating, co-stimulatory, and cytokine signals. While pre-clinical studies assessing CAR-NK cell anti-tumor function have yielded encouraging results against various solid tumor models, many of these systems did not include TME components, and thus indications for the activity of these CAR-NK cells in harsh TMEs is lacking [27,28,29,30,31,32,33,34,35,36,37,38]. Nevertheless, CAR-NK cells have since been employed in active Phase 1/2 clinical trials focused on solid malignancies like glioblastoma, prostate, ovarian, pancreatic, and lung cancer [39,40,41]. Previous trials in patients with solid tumors using CAR-T cells with analogous CAR designs to those being employed in NK cells have not resulted in objective responses. Thus, it can be anticipated that CAR-NK cells similarly need to overcome the hurdles discovered from CAR-T solid tumor trials like tumor antigen down-regulation, tumor heterogeneity, and the immunosuppressive TME [42]. Strategies to overcome these hurdles to improve the efficacy of CAR-NK cell therapy are currently being developed.

CAR-NK cell studies are often performed using CAR constructs with co-stimulatory domains optimized for T-cell activation. Although CD3ζ- and 4-1BB-mediated signaling is conserved between T and NK cells, other commonly used co-stimulatory domains like CD28 are either less effective or completely absent in NK cells [43]. These differences in the induction of activating signals in NK cells would likely lead to sub-optimal CAR-NK cell cytolytic capacity. Endogenous activating receptors on NK cells use adaptor molecules such as CD3ζ, DAP10, and DAP12 to initiate downstream signaling [43]. Li et al. successfully transduced NK cells derived from induced pluripotent stem cell (iPSC) with an anti-mesothelin CAR containing NK cell-centric 2B4 co-stimulatory domain and the NKG2D transmembrane domain (‘NK-centric’ CARs) [44]. In vivo studies using ovarian carcinoma xenografts in immunodeficient mice demonstrated that ‘NK-centric’ CAR transduced NK cells displayed superior anti-tumor activity, reduced tumor burden, and improved mouse survival when compared to NK cells transduced with traditional CARs containing the T cell signaling domain, CD28. Similarly, the NK cell line YTS transduced with CARs containing DAP12 have been utilized for targeting prostate stem cell antigen (PSCA) expressed on tumor cells (PSCA-DAP12 CAR) [45]. PSCA-DAP12 CAR engineered NK cells displayed greater anti-tumor activity compared to non-modified NK cells in PSCA+ tumor xenografts in immunodeficient mice. These studies highlight the importance of utilizing CAR endodomains and transmembrane regions optimized for activity specifically in NK cells to increase the likelihood that these engineered NK cells have heightened activity within solid TMEs.

To improve the in vivo persistence of CAR-NK cells within TMEs containing a highly inhibitory cytokine milieu, NK cells can be engineered to co-express stimulatory cytokine-based transgenes with CARs. Common γ-chain cytokines like IL-2, IL-7, IL-15, and IL-21 are key players in promoting NK cell proliferation and survival [46]. Their incorporation into CAR design may not only promote CAR-NK cell persistence within the TME, but potentially reprogram it to an immune-activating environment to facilitate the infiltration of other immune cells, thereby amplifying anti-tumor immune responses. Liu et al. genetically engineered primary human NK cells to co-express a CD19-directed CAR and IL-15 transgene. These “armored” CAR-transduced NK cells constitutively produced transgenic soluble IL-15 that sustained their growth over a period of 42 days in culture and improved their anti-tumor capacity [47]. However, these studies were limited to lymphoma models and results have not been replicated in a solid tumor model system. Although not NK cell specific, the effects of IL-15 were studied in solid tumor model system using NK-T cells designed to co-express a GD2 antigen specific CAR and IL-15 transgene [48]. The inclusion of IL-15 augmented the therapeutic activity of the GD2 CAR expressing NK-T cells in neuroblastoma xenografts in immunodeficient mice by significantly improving CAR NK-T persistence and improved survival of mice with no observed cytokine-driven toxicity.

The use of CARs not only bolsters NK cell activation against tumor cells but also equips them with the ability to target a wider selection of antigens. This flexibility in CAR targeting can be employed to generate CAR-NK cells that specifically target inhibitory cellular components in the TME to directly delete sources of immunosuppression or utilize TME components to drive CAR expression. One of the main concerns of CAR-based therapy is off-target toxicity since most solid tumors express antigens that are also present at some level on non-malignant tissues. Therefore, strategies are required to ensure that CAR-expressing immune effectors are only active when localized in the tumor tissue. Since hypoxia is a crucial aspect of the TME, utilizing TME hypoxia to drive CAR expression represents an innovative CAR design for improving the therapeutic capacity of CAR therapy while preventing off-target toxicity. A proof-of-concept in vitro study using a lymphoma tumor model demonstrated that a novel CAR construct containing the oxygen-sensitive domain of HIF-1α (HIF-CAR), in which CAR expression was dependent on low concentrations of oxygen in the immediate environment, could limit CAR expression and activity to the TME [49]. While most of these reviewed studies were performed using immune cells other than NK cells, the principles of CAR design used to generate these cell products may be translated to NK cells and used in solid tumor model systems.

### 3.2. Dominant Negative and Switch Receptors

The TME is pervaded by a variety of inhibitory cytokines, including TGF-β which facilitates tumor immunosuppression and supports tumor progression [50]. Elevated TGF-β levels in multiple tumor types correlate with poor prognosis and higher likelihood of recurrence [51,52,53]. TGF-β secreted by tumor cells, as well as by MDSCs, M2 macrophages, and Tregs of the TME, contributes to tumor invasiveness and inhibits anti-tumor response of tumor infiltrating NK cells [14,15,54,55]. TGF-β has been reported to modulate NK cell cytotoxicity by down-regulating the NK cell activating receptors NKG2D and NKp30 as well impacting IFN-γ secretion upon NK activation [56,57,58,59,60]. Thus, to combat this key TME inhibitory mechanism, TGF-β signaling was addressed in the context of NK cell therapy. To improve the clinical efficacy of adoptive NK cell therapy in patients with solid tumors, NK cells have been genetically engineered to express either ‘dominant negative’ or ‘switch receptors’ specific to TGF-β as strategies to overcome TGF-β-mediated inhibition.

Dominant negative receptors lack intracellular signaling domains, and thus do not transduce signals received at the cell surface but can still bind/neutralize the external cytokine. Yvon et al. utilized this concept by engineering a TGF-β receptor II (DNRII) lacking a cytoplasmic domain to improve the anti-tumor activity of cord-blood derived (CB) primary NK cells [61]. CB NK cells transduced with DNRII and expanded using a modified feeder cell line expressing 4-1BBL and membrane bound (mb) IL-21 displayed an activating receptor phenotype similar to that in non-transduced controls. NKD2D and DNAM-1 expression levels on DNRII transduced NK cells in particular were found to be unaffected even in the presence of TGF-β. Although the expansion capacity of DNRII-NK cells was reduced compared to unmodified NK cells, expanded cell numbers were deemed sufficient for clinical use. As a biologic proof-of-concept, phosphorylation of SMAD2 (p-SMAD2) downstream of TGF-β was not detected in DNRII-NK cells as compared to elevated p-SMAD2 in unmodified NK cells. DNRII-NK cells exposed to TGF-β exhibited enhanced cytotoxicity against glioblastoma tumor cells and retained the ability to secrete IFN-γ, perforin, and granzyme-B compared to TGF-β-treated unmodified NK cells, confirming the desired biologic effect. A similar study by Yang et al. was conducted using DNRII-transduced NK-92 cells. DNRII-NK-92 cells were insensitive to TGF-β mediated inhibition with a marked absence of p-SMAD2 and demonstrated enhanced cytolytic activity against lung carcinoma in vitro as well as in a xenograft mouse model compared to that in unmodified NK-92 cells [62].

‘Switch receptors’ and inverted cytokine receptors (ICRs) are chimeric receptors that comprise the extracellular domain of an inhibitory receptor fused to signaling endodomains of an activating receptor. This leads to the conversion of an extracellular inhibitory stimulus to a downstream internal activating signal for cellular activation. Burga et al. assessed the anti-tumor activity of feeder cell expanded CB NK cells transduced with a TGF-β receptor II-based switch receptor containing either a DAP12 domain (NKA) or a Notch minimal regulatory region containing a RELA binding domain (SynNotch) [63]. Both NKA- and SynNotch-transduced NK cells displayed an activation receptor phenotype similar to that of DNRII transduced NK cells and unmodified NK cells. In the presence of TGF-β, both NKA- and SynNotch-NK cells upregulated NKG2D and DNAM-1, and increased levels of phosphorylated Akt compared to DNRII-expressing and unmodified NK cells. Additionally, p-SMAD2/3 was measurable in TGF-β-treated NKA- or SynNotch-NK cells. TGF-β pre-treated NKA- and SynNotch-NK cells exhibited cytotoxicity similar to DNRII-NK cells and enhanced cytotoxicity against neuroblastoma tumor cells compared to unmodified NK cells. Repeated doses of NKA- and SynNotch-NK cells improved the survival of neuroblastoma xenografts compared to unmodified NK cells. However, mice injected with NKA-NK cells had significantly higher survival rates and significantly lower tumor burden compared to both SynNotch- and DNRII NK cells. Additionally, only NKA-NK cells persisted for longer than 60 days in mice, suggesting that coupling the exogenous portion of the TGF-β receptor to DAP12 signaling domain conferred potent internal signals for long-term survival in addition to anti-tumor therapeutic efficacy. A similar study was conducted by Wang et al. who transduced NK-92 cells with switch-receptor comprising the extracellular domain of TGF-β receptor II fused to the intracellular domain of NKG2D (TN) [64]. TGF-β-treated TN-transduced NK-92 cells were found to be resistant to TGF-β downstream signaling as suggested by the absence if intracellular p-SMAD2 compared to empty vector NK-92 controls. Additionally, TN-NK-92 cells expressed higher levels of IFN-γ when stimulated with phorbol myristate acetate (PMA) and ionomycin in the presence of TGF-β. TGF-β-treated TN-NK92 demonstrated enhanced cytotoxicity against hepatocellular carcinoma and prostate tumor cells compared to empty vector NK-92. Incorporation of the TN transgene also improved the homing capacity of NK-92 towards TGF-β secreting tumor cells in vitro. This suggests that the TN transgene may improve the ability of NK cells to infiltrate TGF-β rich tumor microenvironments. Despite promising in vitro data, adoptive transfer of TN-NK-92 cells only moderately improved outcomes in hepatocellular xenografts in athymic nude mice, suggesting that the NKG2D intracellular domain may not be the optimal domain to select for TGF-β-based switch receptor design.

### 3.3. Enhanced NK Receptors and Genetic Deletions

Tumor and inhibitory immune cells of the TME can restrain NK cell anti-tumor responses through a variety of mechanisms. These include the secretion of soluble ligands that block or neutralize NK activating receptor function, matrix metalloproteinases and inhibitory cytokines, and the expression of ligands to inhibitory receptors on infiltrating NK cells. Often times, these tactics employed by the TME result in the down-regulation of activating receptors and/or their adaptor signaling molecules, and upregulation of inhibitory receptors like TIGIT and PD-1, which lead to impaired NK cell cytotoxicity and cytokine/chemokine secretion [54,65,66,67,68,69,70,71,72]. Therefore, strategies to improve NK cell-based therapies against solid tumors have been described that engineer NK cells to express activating receptors resistant to TME induced down-regulation, or NK cells that lack inhibitory receptors.

Parihar et al. approached this challenge by engineering peripheral blood-derived primary NK cells to express a chimeric NKG2D receptor comprising the extracellular and transmembrane domains of NKG2D and a CD3ζ intracellular domain (NKG2D.ζ), thereby circumventing the reliance of NKG2D on DAP10 signaling that is routinely down-modulated within the TME [73]. NKG2D.ζ NK cells expanded using modified K562 feeder line expressing 4-1BBL and mbIL-15 were able to exhibit enhanced cytotoxicity against several solid tumor cell lines expressing NKG2D ligands. In addition, these chimeric NKG2D-expressing NK cells also killed human MDSCs over short- and long-term co-cultures while unmodified NK cells did not. Such cytotoxicity was also exhibited when NKG2D.ζ NK cells were cultured with neuroblastoma patient-derived MDSCs. Importantly, NKG2D.ζ NK cells retained expression of the chimeric receptor in the presence of TGF-β or soluble NKG2D ligands, sMICA and sMICB, suggesting that chimeric NKG2D.ζ was resistant to TME inhibition compared to unmodified NK cells which significantly down-regulated their endogenous NKG2D expression. NKG2D.ζ NK cells injected into NSG mice with TME xenografts containing NKG2D-L+ MDSCs and neuroblastoma tumor cells exhibited enhanced tumor control against MDSC-containing xenografts and successfully improved survival compared to unmodified NK cells. Interestingly, when combined with infusion of GD2 antigen-directed CAR-T cells, NKG2D.ζ NK cells not only enhanced the recruitment of GD2 CAR-T cells to MDSC-containing tumor xenografts, but also improved GD2 CAR-T cell infiltration and anti-tumor activity after NK elimination of intra-tumoral MDSCs. A similar study was conducted using a different version of the NKG2D chimeric receptor which included a DAP10 signaling transgene in addition to the CD3ζ domain (NKG2D.DAP10.ζ) into primary expanded NK cells [74]. Interestingly, NKG2D expression levels in expanded NK cells were higher when DAP10 transgene was present compared to NKG2D.ζ transduced NK cells. NKG2D.DAP10.ζ NK cells consistently exhibited enhanced in vitro cytotoxicity against a broad panel of solid tumor cell lines with a marked increase in IFN-γ secretion compared to mock transduced NK cells. Engagement of the NKG2D.DAP10.ζ receptor with its target activated key DAP10 downstream signaling effectors NF-κβ and AKT. NKG2D.DAP10.ζ NK cell also demonstrated in vivo cytotoxicity in an osteosarcoma xenograft-containing NSG mouse model, resulting in lowered tumor burden compared to mock transduced NK cells.

Xiao et al. designed a chimeric NKG2D comprising only the NKG2D extracellular domain fused to the DAP12 intracellular domain (NKG2D.DAP12) and transduced primary NK cells [75]. NKG2D.DAP12 expression successfully enhanced NK cell cytotoxicity against ovarian, colon, and pharyngeal cancer cell lines in vitro. Repeated infusions of NKG2D.DAP12 NK cells in NSG mice containing colorectal xenografts delayed tumor growth and improved their overall survival compared to mock transduced NK cells. Clinical efficacy of NKG2D.DAP12 NK cells generated from autologous NK cells or from haploidentical donors was assessed in a pilot clinical study comprising three patients with metastatic colorectal cancer. To ease clinical manufacturing by not requiring viral vectors and to ensure transient CAR expression as a means to decrease potential toxicity, NKG2D.DAP12 was introduced into NK cells through mRNA electroporation. Although repeated infusions of NKG2D.DAP12 NK cells controlled malignant ascites in treated patients, best overall response in 1 of 3 patients was stable disease. No severe adverse events were reported in the 4-week toxicity assessment period.

An alternative approach to enhance NK activating receptors for the improvement of NK functions within the TME has focused on engineering NK cells for enhanced antibody-dependent cellular cytotoxicity (ADCC) against tumor targets. Although clinical trials employing tumor-targeting antibodies administered with cytokines, drugs that enhance endogenous NK cell expansion and ADCC activity, or adoptively transferred NK cells, have been reported, improvements in clinical outcomes as compared to administration of antibody alone have been modest [76,77,78,79,80,81,82,83]. This can be partially explained by TME-associated matrix metalloproteinases that cleave Fcγ receptors on NK cells (FcγRIIIa or CD16) and inhibit their ability to engage in ADCC against solid tumors [5]. Snyder et al. sought to enhance the ADCC potential of NK-92 cells and induced pluripotent stem cell-derived NK (iNK) cells by designing a chimeric protein comprising the extracellular portion of CD64, the highest affinity Fcγ receptor, and the transmembrane and intracellular domain of CD16A (CD64/CD16A) [84]. Additionally, the CD64/CD16A fusion receptor lacked the binding site for the matrix metalloproteinase, ADAM17, making it resistant to cleavage in the TME. CD64/CD16A expressed in NK-92 cells and iNK cells facilitated improved recognition and conjugation to HER2+ ovarian tumor cells treated with the anti-HER monoclonal antibody (mAb), trastuzumab, and led to enhanced IFN-γ production by CD64/CD16A-expressing NK cells. Importantly, CD64/CD16A expression was not down-regulated upon antibody-mediated NK cell activation in contrast to the endogenous CD16 molecule, which was down-regulated on the NK cell surface after engagement with trastuzumab.

A similar study was conducted using an engineered high-affinity non-cleavable CD16A receptor (hnCD16A) expressed on iNK cells [85]. hnCD16A was stably expressed and was not down-regulated upon iNK cell activation. hnCD16A-iNK cells exhibited enhanced degranulation and IFN-γ secretion against antibody-treated ovarian and squamous carcinoma cells in vitro compared to unmodified iNK cells. Combination treatment with hnCD16A-iNK cells and anti-HER2 mAb reduced tumor burden and improved survival in mice, demonstrating that hnCD16A mediated a stronger ADCC response than the endogenous CD16 receptor.

In addition to NK cells expressing enhanced activating receptors such as CD16, engineering approaches have also focused on the development of NK cells lacking inhibitory receptors. An approach developed by Pomeroy et al. employed CRISPR/Cas9 technology to genetically abrogate the expression of the inhibitory receptor PD-1 in primary NK cells [86]. Deletion of PD-1 in expanded primary NK cells achieved a knockout (KO) efficiency of 82% and PD-1 KO NK cells could be further expanded to numbers suitable for clinical use. Pre-clinical studies demonstrated that PD-1 KO enhanced the cytotoxicity of NK cells against prostate carcinoma and ovarian carcinoma cell lines in vitro and that a single dose of PD-1 KO NK cells given to mice containing ovarian xenografts successfully reduced tumor burden and moderately improved their survival compared to unmodified NK cells.

### 3.4. NK Checkpoint Inhibitors

While genetic approaches that confer enhanced cytotoxicity and immune-modulating potential within the TME are an attractive option for engineering NK cells, genetic manipulation of NK cells can be technically challenging and thus is not yet readily available at all centers [87,88,89,90]. Alternative strategies to enhance NK functions which do not require genetic modifications and/or robust ex vivo NK cell expansion protocols include development of NK cell-specific checkpoint inhibitors and use of chimeric proteins that co-engage NK cell receptors with tumor targets (i.e., NK cell engagers). While checkpoint inhibitors have primarily been aimed at reinvigorating T cell responses, NK cells are now emerging as a viable target effector for these therapies as NK cells express PD-1, LAG 3, TIM-3 and TIGIT, as well as inhibitory receptors like NKG2A and KIRs [91].

#### 3.4.1. NKG2A

The monoclonal antibody monalizumab, which binds and neutralizes the inhibitory NK receptor, NKG2A, produced robust cytotoxicity against NKG2A ligand-expressing tumor cells and against an antibody-coated squamous cell carcinoma of the head and neck (SCCHN) cell line [92]. Interim results of a Phase II clinical trial of monalizumab plus cetuximab in patients with previously treated squamous cell carcinoma of the head and neck showed a 31% objective response rate (NCT02643550). Most adverse events (93%) were grade 1–2 and were easily managed [92]. Most common monalizumab-related adverse events were fatigue (17%), pyrexia (13%), and headache (10%). These results suggest that the ligands for NKG2A expressed within solid TMEs inhibit endogenous NK cells, and that this inhibition may be reversed by ‘unleashing’ the NK cell checkpoint NKG2A. Efforts to determine whether the NKG2A-ligand axis may also similarly inhibit adoptively transferred NK cells are underway.

#### 3.4.2. KIRs

Solid tumors evade NK cell immunosurveillance by upregulating distinct HLA class I allotypes specific to inhibitory KIRs on NK cells [93,94]. Sola et al. first reported the effectiveness of the anti-KIR2DL1/2/3 monoclonal antibody lirilumab in RAG-1 deficient mice containing HLA-C expressing B cell lymphoma xenografts [95]. NK cells that expressed human inhibitory KIR2DL3 were generated in this background (KIRtgRAG mice). Lirilumab infusion significantly improved the survival of KIRtgRAG mice in a dose dependent manner. A phase I study showed that infusion of lirilumab did not result in severe adverse events or dose-limiting toxicity in patients with solid tumors [96]. The most frequent adverse events recorded were grade 1–2 pruritus (19%), asthenia (16%), fatigue (14%), infusion-related reactions (14%) and headache (11%). However, no objective responses were noted. A transient decrease in peripheral NK cell numbers was noted after the first cycle of treatment. While no significant changes in CD69 expression were noted in circulating KIR+ NK cells after treatment, further assessments of NK cell function and phenotype before and after treatment were not conducted within the context of this trial. Currently, the clinical efficacy of lirilumab in combination with nivolumab and ipilimumab is being evaluated in patients with advanced or metastatic solid tumors (NCT03203876).

#### 3.4.3. TIM-3

The immune checkpoint TIM-3 is constitutively expressed on human NK cells and binds ligands like galectin-9 whose expression is upregulated in the TME and plays a role in suppressing NK cell-mediated cytotoxicity in vitro [97,98]. TIM-3 upregulation has also been linked to NK cell dysfunction and poor prognosis in patients with bladder cancer and advanced melanoma [99,100]. Thus, blocking TIM-3 may be a suitable therapeutic strategy for enhancement of NK cell function. Pre-clinical studies have demonstrated that antibody blockade of TIM-3 on peripheral blood NK cells isolated from patients with lung adenocarcinoma moderately improved their cytotoxicity and IFN-γ production in vitro [101]. A similar study by da Silva et al. showed antibody blockade of TIM-3 rescued the anti-tumor activity of functionally impaired peripheral blood NK cells derived from patients with metastatic melanoma against melanoma tumor line targets [100]. Additionally, Farkas et al. demonstrated that blockade of TIM-3 enhanced the effector function of bladder cancer patient-derived NK cells upon IL-15 stimulation [99]. However, these effects could not be replicated in intra-tumoral NK cells, which suggests that TIM-3 blockade alone may not be sufficient to fully rescue NK cells within the TME where multiple immune checkpoint ligands are expressed [102].

#### 3.4.4. TIGIT

TIGIT, like TIM-3, is constitutively expressed on human NK cells and has been reported to suppress NK cell IFN-γ production in chronic viral conditions [103]. Importantly, TIGIT is upregulated on intra-tumoral NK cells and is capable of binding the poliovirus receptor (CD155) and nectin-2 (CD112), both of which are expressed in solid tumor tissues [104]. Zhang et al. demonstrated that TIGIT blockade in immunocompetent tumor-bearing mice improved their survival. Importantly, the therapeutic effect of TIGIT antibody blockade was found to be NK cell-dependent as antibody-mediated NK cell depletion in mice 24 h prior to TIGIT blockade abrogated the positive effects of TIGIT antibody treatment [104]. Similar studies in human peripheral blood NK cells showed that TIGIT blockade enhanced their anti-tumor activity against soft-tissue sarcoma and ovarian carcinoma tumor targets in vitro, and significantly, reduced tumor burden of mice with ovarian carcinoma xenografts [105,106]. These encouraging pre-clinical results have led to the initiation of multiple clinical trials using monoclonal anti-TIGIT antibodies alone or in combination with other checkpoint inhibitors (NCT02913313, NCT04570839].

### 3.5. Engagers

Other antibody-based molecules that have been developed to improve NK cell anti-tumor activity are Bispecific Natural Killer Engagers (BiKEs) and Trispecific Natural Killer Engagers (TriKEs). BiKEs typically comprise two monoclonal antibody fragments, one specific to endogenous CD16 on NK cells, and the other specific to a tumor-associated antigen, connected by a linker. TriKEs utilize a similar design, but also include a stimulatory cytokine such as IL-15 or IL 21 meant to improve NK expansion, viability, and function within a TME. While both systems promoted NK cell cytotoxicity against tumor via CD16 activation, the TriKE system also improved NK cell survival. Both systems also enhanced NK cell cytotoxicity against a component of the TiME, CD33+ MDSCs [107,108,109]. TriKEs also improved NK cell survival and proliferation capacity compared to BiKEs in tumor bearing NSG mice [109]. Versions of these agents are currently being tested in the context of clinical trials (NCT03214666; NCT00560794). Additionally, TriKEs have now been adapted to target antigens expressed on solid tumors. Vallera et al. developed a TriKE containing an IL-15 moiety which targeted a solid tumor-associated antigen B7-H3 (cam1615B7H3) [110]. cam1615B7H3 induced NK cell expansion and cytolytic activity and led to reduced tumor burden in mice containing B7-H3 expressing ovarian carcinoma xenografts when compared to NK cells that only received infusions of IL-15. A similar study by Schmohl et al. demonstrated that EpCAM targeted TriKEs could improve NK cell expansion, ADCC and survival against EpCAM+ colorectal carcinoma cells in vitro [111].

## 4. Discussion

Previous reviews have focused on the utility of human NK cells as a cell therapeutic platform for the treatment of cancer [41,112,113]. In this review, we specifically focused on recent strategies employed to enhance NK cell function within inhibitory tumor microenvironments, important next steps in utilizing NK cells for the effective treatment of solid tumors. We have detailed the design and implementation of various genetic engineering and antibody-based approaches to improve the efficacy of NK cell-based therapies in the TME of patients with solid tumors. Many adoptive NK cell therapeutic platforms presented in this review successfully demonstrated anti-tumor activity in pre-clinical in vitro and in vivo studies (Table 1).

Pre-clinical and clinical studies require large numbers of NK cells for experimentation and patient infusion, respectively. Since NK cells comprise a small fraction of the lymphocytic population in peripheral blood (1–20%), strategies have been developed to either enrich them from apheresis products or rapidly expand peripheral blood or iPSC-derived NK cells to large numbers using irradiated feeder cell systems, IL-2, or feeder- and serum-free techniques [114,115,116,117]. These strategies have resulted in expansion of NK cells to numbers that allow for clinical use. Alternatively, NK cell lines such as YTS, NK-92, NKL, and HANK-1 can be used for therapeutic purposes due to their tremendous expansion potential and low cytotoxic and phenotypic variabilities [118,119]. However, the NK-92 cell line lacks the low affinity Fcγ receptor rendering it incapable of initiating ADCC [120]. Additionally, irradiation of NK-92 cells prior to infusion results in shortened survival in vivo and thus a priori requirement for multiple large dose infusions [121]. Despite recent success in NK cell expansion methods, the consequences of various expansion protocols on resultant NK cell phenotypes that may influence their functions within solid TMEs have not been reported and this remains an important question in the field. For example, whether NK cells expanded with protocols that utilize IL-2 vs. IL-15 vs. IL-21 as cytokine co-stimulation differentially express inhibitory receptors such as NKG2A or PD-1, whose ligands are overexpressed within solid TMEs [122,123].

While the various NK cell sources including peripheral blood, cord blood, and iPSCs have proven to be amenable to genetic modification, the selection of suitable transduction methods for efficient and optimal transgenic expression in these cells is an obstacle that needs to be overcome. Lentiviral and retroviral transduction systems provide stable gene expression but run the risk of insertional mutagenesis and require significant regulatory considerations/evaluations [87,124]. Electroporation-based systems for transfer of genetic material have been explored as a potential alternative. The system is safe and provides relatively modest genetic material transfer efficiencies depending on the NK cells and expansion systems utilized. However, gene expression is transient due to eventual degradation of encoding mRNA [88,124].

CAR-NK cells are a promising option as a therapeutic platform and CAR-induced activation has shown to result in heightened intracellular down-stream signaling which supports canonical activation pathways leading to CAR-mediated cytotoxicity [125]. Historically, primary NK cell cytotoxicity and pro-inflammatory cytokine secretion is thought to be tied to the intensity of surface CD56 expression (CD56bright and CD56dim) although that notion is now being challenged [126,127,128]. While CD56bright NK cells have been associated with survival in melanoma patients, their role in the efficacy of cellular therapies is unknown [129]. Still, ex vivo expansion of primary NK cells for CAR-NK cell therapy generates a ‘hybrid’ population of activated NK cells with both high expression of CD56 and CD16 capable of exhibiting both cytotoxic and cytokine-producing effector functions with no apparent distinction based strictly on CD56 expression [130]. Recent reports suggest a role for CD56 in immune synapse formation between NK KIR and targets cells, suggesting CD56’s importance in NK cytotoxic function. However, this biology has not been confirmed in ex vivo manipulated NK cells [128]. Additionally, to improve in vivo persistence, CAR-NK cells have been engineered to express IL-15 which may prove useful in overcoming the suppressive TME [47]. Alternatively, dominant negative and switch receptors aim to either blunt inhibitory signals or convert inhibitory stimuli to activating intracellular signals that improve NK cell cytotoxicity. Switch receptors designed to be specific towards ligands of inhibitory receptors on T cells may also be applied to NK cells which share some of the same inhibitory receptors [131,132]. However, it is unclear whether these receptors, which typically target a single inhibitory molecule, will improve NK cell anti-tumor activity in the context of a TME capable of enforcing suppression through multiple inhibitory molecules. Additionally, the efficacy of such receptors is largely dependent on the availability of the specific inhibitory molecule in the TME. Conversely, continuous activation signals generated through switch receptors and constitutive cytokine transgenes run the risk of initiating activation induced cell death (AICD). It would be beneficial to optimize the design of switch receptors by assessing multiple extracellular and intracellular domain combinations to not only determine their ability to transduce activating signals but also assess their propensity to initiate AICD in NK cells.

Modifications to endogenous activating receptors have largely focused on NKG2D and CD16 to prevent their down-regulation or cleavage in the TME. However, solid tumors and TME components are capable of shedding NKG2D ligands [133]. Additionally, the efficacy of therapies which rely on the administration of therapeutic antibodies is handicapped in the TME due to antibody ‘blocking’ by stromal barriers and antibody trapping by Fc receptors of resident MDSCs and M2 macrophages [134,135,136]. Since NK cells also express natural cytotoxicity receptors (NCRs) that recognize ligands on tumor targets and mediate activating signals, modification of NK cells with engineered NCR transgenes in combination with either modified NKG2D or enhanced CD16 receptors may lead to a more robust NK cell therapy and improve anti-tumor activity.

The continued development of CRISPR/cas9 technology has led to its use as an efficient and potent gene-editing tool in many cell types, including human NK cells [86,137]. While many technical and biological barriers remain such as gRNA design, off-target effects, and post-electroporation NK cell viability, CRISPR/cas9 systems show potential to improve NK cell immunotherapy due to the flexibility of the delivery system as well as a highly efficient gene-editing capacity [89]. Another exciting approach that is being considered is the incorporation of synthetic biological systems to ‘sense and process’ external signals from cell-specific and disease markers [138]. This would lead to greater command over temporal and therapeutic functionality of the therapy making them safer and more effective.

Not surprisingly, approaches to improve the efficacy of cell therapy in the TME have not been limited to NK cells. For example, multiple studies have attempted to utilize CAR-T cells to eliminate immunosuppressive cancer associated fibroblasts by targeting fibroblast activating protein (FAP) [139,140,141]. Unfortunately, FAP directed CAR-T cells have demonstrated the potential for toxicity due to the low level expression of FAP on normal tissue. The use of CAR-NK cells may be a viable alternative due to their shorter life span that limits the number and duration of adverse reactions in normal tissue [39]. In addition, as opposed to MHC-restricted T cells, NK cell activity is modulated by a combination of activating and inhibitory receptors, such as those recognizing MHC-I, that alter the cytotoxic response when encountering normal or transformed tissue [142]. Consequently, NK cells have an overall greater mean time to target cell lysis in part due to the multiple signals needed for a subsequent lytic response [143,144,145]. CAR-T cells have also been modified to express additional factors like scFVs and chemokine receptors to improve cytotoxicity in the TME [146,147,148]. CAR-NK cells have also been genetically modified to express the chemokine receptor CXCR1 leading to improved trafficking and anti-tumor responses in ovarian carcinoma xenografts in mice [149]. The development of novel strategies as well as the application of existing strategies against unique targets informs the development of more efficacious NK cell therapies in the TME.

## 5. Conclusions and Future Considerations

The idea of engineered and non-engineered NK cell-based immunotherapy has steadily gained steam, culminating in successful application in patients with hematological malignancies. However, solid tumors present a unique and difficult challenge due to the inhibitory TME. These unique challenges are not fully represented in published studies assessing human NK cell anti-tumor responses as most pre-clinical models only employ tumor cells (e.g., traditional xenografts in immunodeficient mice). It would be beneficial to either incorporate TME components in pre-clinical studies of adoptive human NK cell therapies to better predict efficacy in patients and to discern how various TME components impact the anti-tumor response, or to utilize immunocompetent models that contain a complete endogenous TME (although the latter would preclude testing of human cell products). Such experimental systems are now gaining popularity when testing anti-tumor responses of immune cells. Three-dimensional tumor spheroid in vitro cultures that incorporate TME components including stromal cells, extracellular matrix proteins, and immune infiltrates are able to mimic TME spatial architecture, secretion of soluble factors, and gene expression patterns [150]. However, they are devoid of ability to assess cell homing, intra-tumoral infiltration, and compartmental expansion. In vivo models that utilize xenografts containing immune microenvironment components such as human MDSCs, as well as humanized mouse models, can simulate the crosstalk between tumor cells and inhibitory immune cells when assessing anti-tumor efficacy of human NK cell-based therapies [73,151,152,153].

Ex vivo expansion of NK cells leads to upregulation of both activating and inhibitory receptors [116,117]. However, the significance of the differences in this upregulation between expansion/activation systems on NK cell cytotoxicity within suppressive TMEs in vivo cannot be assessed as no direct comparison of the NK cell anti-tumor function in the presence of TME components has been made. Such experimentation would aid the development of expansion techniques that generate NK cells capable of potent and durable tumor elimination within an immunosuppressive TME.

Checkpoint blockade therapies targeting inhibitory receptors on immune cells have shown encouraging pre-clinical results as well as satisfactory safety profiles in patients in clinical trials [154]. However, their efficacy has been limited to a small subset of patients and to successful stemming of disease progression, not necessarily disease eradication [92,96]. Thus, effective monotherapy targeting NK cell checkpoints may be challenging since NK cells routinely express multiple inhibitory receptors that check uncontrolled NK activation. Thus, combinations of various checkpoint inhibitors will likely be required to fully mobilize NK cells in the TME. Although many immune checkpoint receptors have been correlated with NK cell dysfunction in solid tumors, comprehensive analyses of their functionality in suppressing of NK cell activity in the TME is limited. Therefore, screening of NK cell phenotypes based on tumor type, tumor stage, and therapeutic setting will be required, not only for the discovery of novel biomarkers, but to also assist clinicians in the selection of suitable therapies for each individual patient.

Aspects of innate NK cell biology may also be exploited to improve NK cell-based therapies. Therapies that harness the cytotoxic capacity of NK cells to mediate ADCC have been described in this review. NK cells are also an important source of chemokines that may promote the trafficking of other immune cells to the TME [155,156,157]. In fact, Parihar et al. reported that NKG2D.ζ NK cell-derived chemokines were able to recruit subsequently infused GD2 CAR-T cells to tumor sites [73]. Studies have also aimed to improve NK cell immunosuppression by targeting the inhibitor metabolite adenosine and the enzyme CD73 that generates it, and monocyte-derived reactive oxygen species via antibody blockade and enzyme inhibitors [158,159,160,161]. Interestingly, tumor infiltrating NK cells upon engagement with 4-1BBL expressing tumors upregulate surface CD73 expression and acquire immunosuppressive properties via STAT3 resulting in IL-10 and TGF-β production [162]. Additionally, CD73+ NK cells are capable of secreting CD73 into the extracellular space which may act on the abundant levels of ATP in the TME to generate additional adenosine, further inhibiting NK cell activity. Therapies that target CD73 or STAT3 may prevent this transcriptional reprogramming of NK cells to maintain its cytolytic state. Finally, recent research has linked NK cell dysfunction to impaired metabolism leading to blunted anti-tumor responses [163,164,165]. The TME plays a significant role in this process [166]. Therapies to disrupt tumor metabolism are currently being tested in clinical trials or clinically approved [167,168,169]. However, these therapies inhibit shared metabolic pathways in NK cells, and also lead to reduced NKG2D ligand expression in tumor cells [170,171]. It would be important to develop strategies to improve NK cell cytotoxicity and proliferation by enhancing the NK cell-specific metabolic programs that control them.

NK cells are an important wing of the anti-tumor response. Uncovering the mechanisms behind their reduced activity in solid TMEs will aid the conceptualization of new therapeutic modalities using NK cells. Additionally, optimization of NK cell ex vivo expansion and in vitro differentiation technologies, along with the implementation of novel cellular and non-cellular approaches detailed herein, would enhance the application of NK cell-based therapies in the clinic. Ultimately, a combination of design and implementation strategies will allow NK cells to become more efficacious for the treatment of solid tumors where tumor microenvironments significantly impede current approaches.

## Figures and Tables

**Table 1:** NK cell therapeutic strategies. Summary of design and implementation strategies and approaches to augment therapeutic responses of NK cells in the solid tumor microenvironment.

Design Strategy	Target	Tumor Model	Origin of NK Cell	Reference
NK-Centric CAR	Mesothelin	Ovarian carcinoma	iPSC-derived NK cells	[44]
	PSCA	Kidney cell transplant	YTS	[45]
Armored CAR	GD2	Neuroblastoma	NK-T cells	[48]
Inducible CAR	CD19	Lymphoma	T cells	[49]
Dominant Negative Receptors	TGF-β	Glioblastoma	Cord blood-derived NK cells	[61]
	TGF-β	Lung carcinoma	NK-92	[62]
Switch Receptors	TGF-β	Neuroblastoma Hepatocellular carcinoma, Prostate carcinoma	Cord blood-derived NK cells	[63]
	TGF-β		NK-92	[64]
Chimeric NK cell receptors	NKG2DL	Neuroblastoma	Primary NK cells	[73]
	NKG2DL	Osteosarcoma	Primary NK cells	[74]
	NKG2DL	Colorectal carcinoma	Primary NK cells	[75]
Enhanced Fcγ receptors	CD64/CD16A	Ovarian carcinoma	iPSC-derived NK cells, NK-92	[84]
	CD16A	Ovarian carcinoma	iPSC-derived NK cells	[85]
Genetic Deletions	PD-1	Ovarian carcinoma	Primary NK cells	[86]
Checkpoint Blockade	NKG2A	Head and neck carcinoma	-	[92]
	TIM-3	Lung adenocarcinoma, Melanoma	-	[100,101]
	TIGIT	Ovarian carcinoma, Ewing’s sarcoma, leiomyosarcoma	-	[105,106]
BiKEs and TriKEs	CD33	Myelodysplastic syndrome	-	[107,108]
	CD33	Acute myeloid leukemia	-	[109]
	B7-H3	Ovarian carcinoma	-	[110]
	EpCAM	Colorectal carcinoma	-	[111]

## References

[1] Liu E., Marin D., Banerjee P., Macapinlac H.A., Thompson P., Basar R., Kerbauy L.N., Overman B., Thall P., Kaplan M. (2020). Use of CAR-Transduced Natural Killer Cells in CD19-Positive Lymphoid Tumors. N. Engl. J. Med..

[2] Brudno J.N., Kochenderfer J.N. (2019). Recent advances in CAR T-cell toxicity: Mechanisms, manifestations and management. Blood Rev..

[3] Smith M., Zakrzewski J., James S., Sadelain M. (2018). Posttransplant chimeric antigen receptor therapy. Blood.

[4] Mitsiades N., Yu W.H., Poulaki V., Tsokos M., Stamenkovic I. (2001). Matrix metalloproteinase-7-mediated cleavage of Fas ligand protects tumor cells from chemotherapeutic drug cytotoxicity. Cancer Res..

[5] Rossi G.R., Trindade E.S., Souza-Fonseca-Guimaraes F. (2020). Tumor Microenvironment-Associated Extracellular Matrix Components Regulate NK Cell Function. Front. Immunol..

[6] Chakravarthy A., Khan L., Bensler N.P., Bose P., De Carvalho D.D. (2018). TGF-β-associated extracellular matrix genes link cancer-associated fibroblasts to immune evasion and immunotherapy failure. Nat. Commun..

[7] Clark R.A.F., McCoy G.A., Folkvord J.M., McPherson J.M. (1997). TGF-β1 stimulates cultured human fibroblasts to proliferate and produce tissue-like fibroplasia: A fibronectin matrix-dependent event. J. Cell. Physiol..

[8] Veglia F., Perego M., Gabrilovich D.I. (2018). Myeloid-derived suppressor cells coming of age. Nat. Immunol..

[9] Gabrilovich D.I., Nagaraj S. (2009). Myeloid-derived suppressor cells as regulators of the immune system. Nat. Rev. Immunol..

[10] Marvel D., Gabrilovich D.I. (2015). Myeloid-derived suppressor cells in the tumor microenvironment: Expect the unexpected. J. Clin. Investig..

[11] Bruno A., Mortara L., Baci D., Noonan D.M., Albini A. (2019). Myeloid Derived Suppressor Cells Interactions With Natural Killer Cells and Pro-angiogenic Activities: Roles in Tumor Progression. Front. Immunol..

[12] Li H., Han Y., Guo Q., Zhang M., Cao X. (2009). Cancer-expanded myeloid-derived suppressor cells induce anergy of NK cells through membrane-bound TGF-β1. J. Immunol..

[13] Burga R.A., Thorn M., Point G.R., Guha P., Nguyen C.T., Licata L.A., DeMatteo R.P., Ayala A., Espat N.J., Junghans R.P. (2015). Liver myeloid-derived suppressor cells expand in response to liver metastases in mice and inhibit the anti-tumor efficacy of anti-CEA CAR-T. Cancer Immunol. Immunother..

[14] Kumar V., Patel S., Tcyganov E., Gabrilovich D.I. (2016). The Nature of Myeloid-Derived Suppressor Cells in the Tumor Microenvironment. Trends Immunol..

[15] Liu Z., Kuang W., Zhou Q., Zhang Y. (2018). TGF-β1 secreted by M2 phenotype macrophages enhances the stemness and migration of glioma cells via the SMAD2/3 signalling pathway. Int. J. Mol. Med..

[16] Nuñez S.Y., Ziblat A., Secchiari F., Torres N.I., Sierra J.M., Iraolagoitia X.L.R., Araya R.E., Domaica C.I., Fuertes M.B., Zwirner N.W. (2017). Human M2 Macrophages Limit NK Cell Effector Functions through Secretion of TGF-β and Engagement of CD85j. J. Immunol..

[17] Qian B., Deng Y., Im J.H., Muschel R.J., Zou Y., Li J., Lang R.A., Pollard J.W. (2009). A Distinct Macrophage Population Mediates Metastatic Breast Cancer Cell Extravasation, Establishment and Growth. PLoS ONE.

[18] Smyth M.J., Teng M.W.L., Swann J., Kyparissoudis K., Godfrey D.I., Hayakawa Y. (2006). CD4+CD25+ T Regulatory Cells Suppress NK Cell-Mediated Immunotherapy of Cancer. J. Immunol..

[19] Chang W.-C., Ling-Hui C., Wen-Chun C., Huang P.-S., Sheu B.-C., Huang S.-C. (2016). Regulatory T Cells Suppress Natural Killer Cell Immunity in Patients With Human Cervical Carcinoma. Int. J. Gynecol. Cancer.

[20] Janco J.M.T., Lamichhane P., Karyampudi L., Knutson K.L. (2015). Tumor-Infiltrating Dendritic Cells in Cancer Pathogenesis. J. Immunol..

[21] Harimoto H., Shimizu M., Nakagawa Y., Nakatsuka K., Wakabayashi A., Sakamoto C., Takahashi H. (2013). Inactivation of tumor-specific CD8+ CTLs by tumor-infiltrating tolerogenic dendritic cells. Immunol. Cell Biol..

[22] Scarlett U.K., Rutkowski M.R., Rauwerdink A.M., Fields J., Escovar-Fadul X., Baird J., Cubillos-Ruiz J.R., Jacobs A.C., Gonzalez J.L., Weaver J. (2012). Ovarian cancer progression is controlled by phenotypic changes in dendritic cells. J. Exp. Med..

[23] Schwartz M., Zhang Y., Rosenblatt J.D. (2016). B cell regulation of the anti-tumor response and role in carcinogenesis. J. Immunother. Cancer.

[24] Melaiu O., Lucarini V., Cifaldi L., Fruci D. (2020). Influence of the Tumor Microenvironment on NK Cell Function in Solid Tumors. Front. Immunol..

[25] Nayyar G., Chu Y., Cairo M.S. (2019). Overcoming Resistance to Natural Killer Cell Based Immunotherapies for Solid Tumors. Front. Oncol..

[26] Hu J., Bernatchez C., Zhang L., Xia X., Kleinerman E.S., Hung M.-C., Hwu P., Li S. (2017). Induction of NKG2D Ligands on Solid Tumors Requires Tumor-Specific CD8+ T Cells and Histone Acetyltransferases. Cancer Immunol. Res..

[27] Schönfeld K., Sahm C., Zhang C., Naundorf S., Brendel C., Odendahl M., Nowakowska P., Bönig H., Köhl U., Kloess S. (2015). Selective Inhibition of Tumor Growth by Clonal NK Cells Expressing an ErbB2/HER2-Specific Chimeric Antigen Receptor. Mol. Ther..

[28] Zhang C., Oberoi P., Oelsner S., Waldmann A., Lindner A., Tonn T., Wels W.S. (2017). Chimeric Antigen Receptor-Engineered NK-92 Cells: An Off-the-Shelf Cellular Therapeutic for Targeted Elimination of Cancer Cells and Induction of Protective Antitumor Immunity. Front. Immunol..

[29] Zhang C., Burger M.C., Jennewein L., Genßler S., Schönfeld K., Zeiner P., Hattingen E., Harter P.N., Mittelbronn M., Tonn T. (2015). ErbB2/HER2-Specific NK Cells for Targeted Therapy of Glioblastoma. J. Natl. Cancer Inst..

[30] Chen X., Han J., Chu J., Zhang L., Zhang J., Chen C., Chen L., Wang Y., Wang H., Yi L. (2016). A combinational therapy of EGFR-CAR NK cells and oncolytic herpes simplex virus 1 for breast cancer brain metastases. Oncotarget.

[31] Murakami T., Nakazawa T., Natsume A., Nishimura F., Nakamura M., Matsuda R., Omoto K., Tanaka Y., Shida Y., Park Y.-S. (2018). Novel Human NK Cell Line Carrying CAR Targeting EGFRvIII Induces Antitumor Effects in Glioblastoma Cells. Anticancer. Res..

[32] Han J., Chu J., Chan W.K., Zhang J., Wang Y., Cohen J.B., Victor A., Meisen W.H., Kim S.-H., Grandi P. (2015). CAR-Engineered NK Cells Targeting Wild-Type EGFR and EGFRvIII Enhance Killing of Glioblastoma and Patient-Derived Glioblastoma Stem Cells. Sci. Rep..

[33] Sahm C., Schönfeld K., Wels W. (2012). Expression of IL-15 in NK cells results in rapid enrichment and selective cytotoxicity of gene-modified effectors that carry a tumor-specific antigen receptor. Cancer Immunol. Immunother..

[34] Richards R.M., Sotillo E., Majzner R.G. (2018). CAR T Cell Therapy for Neuroblastoma. Front. Immunol..

[35] Esser R., Müller T., Stefes D., Kloess S., Seidel D., Gillies S.D., Aperlo-Iffland C., Huston J.S., Uherek C., Schönfeld K. (2012). NK cells engineered to express a GD2-specific antigen receptor display built-in ADCC-like activity against tumour cells of neuroectodermal origin. J. Cell. Mol. Med..

[36] Kailayangiri S., Altvater B., Spurny C., Jamitzky S., Schelhaas S., Jacobs A.H., Wiek C., Roellecke K., Hanenberg H., Hartmann W. (2017). Targeting Ewing sarcoma with activated and GD2-specific chimeric antigen receptor-engineered human NK cells induces upregulation of immune-inhibitory HLA-G. OncoImmunology.

[37] Specht J.M., Lee S., Turtle C., Berger C., Veatch J., Gooley T., Mullane E., Chaney C., Riddell S., Maloney D.G. (2018). Phase I study of immunotherapy for advanced ROR1+ malignancies with autologous ROR1-specific chimeric antigen receptor-modified (CAR)-T cells. J. Clin. Oncol..

[38] Park H., Awasthi A., Ayello J., Chu Y., Riddell S., Rosenblum J., Lee D.S., Cairo M.S. (2017). ROR1-Specific Chimeric Antigen Receptor (CAR) NK Cell Immunotherapy for High Risk Neuroblastomas and Sarcomas. Biol. Blood Marrow Transpl..

[39] Xie G., Dong H., Liang Y., Ham J.D., Romee R., Chen J. (2020). CAR-NK cells: A promising cellular immunotherapy for cancer. EBioMedicine.

[40] Wang W., Jiang J., Jiang J. (2020). CAR-NK for tumor immunotherapy: Clinical transformation and future prospects. Cancer Lett..

[41] Oh S., Lee J.H., Kwack K., Choi S.-W. (2019). Natural Killer Cell Therapy: A New Treatment Paradigm for Solid Tumors. Cancers.

[42] Rodriguez-Garcia A., Palazon A., Noguera-Ortega E., Powell D.J.J., Guedan S. (2020). CAR-T Cells Hit the Tumor Microenvironment: Strategies to Overcome Tumor Escape. Front. Immunol..

[43] Pfefferle A., Huntington N.D. (2020). You Have Got a Fast CAR: Chimeric Antigen Receptor NK Cells in Cancer Therapy. Cancers.

[44] Li Y., Hermanson D.L., Moriarity B.S., Kaufman D.S. (2018). Human iPSC-Derived Natural Killer Cells Engineered with Chimeric Antigen Receptors Enhance Anti-tumor Activity. Cell Stem Cell.

[45] Töpfer K., Cartellieri M., Michen S., Wiedemuth R., Müller N., Lindemann D., Bachmann M., Füssel M., Schackert G., Temme A. (2015). DAP12-Based Activating Chimeric Antigen Receptor for NK Cell Tumor Immunotherapy. J. Immunol..

[46] Meazza R., Azzarone B., Orengo A.M., Ferrini S. (2011). Role of Common-Gamma Chain Cytokines in NK Cell Development and Function: Perspectives for Immunotherapy. J. Biomed. Biotechnol..

[47] Liu E., Tong Y., Dotti G., Shaim H., Savoldo B., Mukherjee M., Orange J., Wan X., Lu X., Reynolds A. (2018). Cord blood NK cells engineered to express IL-15 and a CD19-targeted CAR show long-term persistence and potent antitumor activity. Leukemia.

[48] Xu X., Huang W., Heczey A., Liu D., Guo L., Wood M., Jin J., Courtney A.N., Liu B., Di Pierro E.J. (2019). NKT Cells Coexpressing a GD2-Specific Chimeric Antigen Receptor and IL15 Show Enhanced In Vivo Persistence and Antitumor Activity against Neuroblastoma. Clin. Cancer Res..

[49] Juillerat A., Marechal A., Filhol J.M., Valogne Y., Valton J., Duclert A., Duchateau P., Poirot L. (2017). An oxygen sensitive self-decision making engineered CAR T-cell. Sci. Rep..

[50] Yang L., Pang Y., Moses H.L. (2010). TGF-β and immune cells: An important regulatory axis in the tumor microenvironment and progression. Trends Immunol..

[51] Friedman E., Gold L.I., Klimstra D., Zeng Z.S., Winawer S., Cohen A. (1995). High levels of transforming growth factor beta 1 correlate with disease progression in human colon cancer. Cancer Epidemiol. Biomark. Prev..

[52] Park H., Bang J., Nam A., Park J.E., Jin M.H., Bang Y., Oh D.-Y. (2019). The prognostic role of soluble TGF-beta and its dynamics in unresectable pancreatic cancer treated with chemotherapy. Cancer Med..

[53] An Y., Gao S., Zhao W.-C., Qiu B.-A., Xia N.-X., Zhang P.-J., Fan Z. (2018). Transforming growth factor-β and peripheral regulatory cells are negatively correlated with the overall survival of hepatocellular carcinoma. World J. Gastroenterol..

[54] Lazarova M., Steinle A. (2019). Impairment of NKG2D-Mediated Tumor Immunity by TGF-β. Front. Immunol..

[55] Dahmani A., Delisle J.-S. (2018). TGF-β in T Cell Biology: Implications for Cancer Immunotherapy. Cancers.

[56] Trotta R., Col J.D., Yu J., Ciarlariello D., Thomas B., Zhang X., Allard J., Wei M., Mao H., Byrd J.C. (2008). TGF-β Utilizes SMAD3 to Inhibit CD16-Mediated IFN-γ Production and Antibody-Dependent Cellular Cytotoxicity in Human NK Cells1. J. Immunol..

[57] Kopp H.-G., Placke T., Salih H.R. (2009). Platelet-Derived Transforming Growth Factor- Down-Regulates NKG2D Thereby Inhibiting Natural Killer Cell Antitumor Reactivity. Cancer Res..

[58] Castriconi R., Cantoni C., Della Chiesa M., Vitale M., Marcenaro E., Conte R., Biassoni R., Bottino C., Moretta L., Moretta A. (2003). Transforming growth factor 1 inhibits expression of NKp30 and NKG2D receptors: Consequences for the NK-mediated killing of dendritic cells. Proc. Natl. Acad. Sci. USA.

[59] Lee J.-C., Lee K.-M., Kim D.-W., Heo D.S. (2004). Elevated TGF-β1 Secretion and Down-Modulation of NKG2D Underlies Impaired NK Cytotoxicity in Cancer Patients. J. Immunol..

[60] Friese M.A., Wischhusen J., Wick W., Weiler M., Eisele G., Steinle A., Weller M. (2004). RNA Interference Targeting Transforming Growth Factor-β Enhances NKG2D-Mediated Antiglioma Immune Response, Inhibits Glioma Cell Migration and Invasiveness, and Abrogates TumorigenicityIn vivo. Cancer Res..

[61] Yvon E.S., Burga R., Powell A., Cruz C.R., Fernandes R., Barese C., Nguyen T., Abdel-Baki M.S., Bollard C.M. (2017). Cord blood natural killer cells expressing a dominant negative TGF-β receptor: Implications for adoptive immunotherapy for glioblastoma. Cytotherapy.

[62] Yang B., Liu H., Shi W., Wang Z., Sun S., Zhang G., Hu Y., Liu T., Jiao S. (2013). Blocking transforming growth factor-β signaling pathway augments antitumor effect of adoptive NK-92 cell therapy. Int. Immunopharmacol..

[63] Burga R.A., Yvon E., Chorvinsky E., Fernandes R., Cruz C.R.Y., Bollard C.M. (2019). Engineering the TGFβ Receptor to Enhance the Therapeutic Potential of Natural Killer Cells as an Immunotherapy for Neuroblastoma. Clin. Cancer Res..

[64] Wang Z., Guo L., Song Y., Zhang Y., Lin D., Hu B., Mei Y., Sandikin D., Liu H. (2017). Augmented anti-tumor activity of NK-92 cells expressing chimeric receptors of TGF-βR II and NKG2D. Cancer Immunol. Immunother..

[65] Raffaghello L., Prigione I., Airoldi I., Camoriano M., Levreri I., Gambini C., Pende D., Steinle A., Ferrone S., Pistoia V. (2004). Downregulation and/or Release of NKG2D Ligands as Immune Evasion Strategy of Human Neuroblastoma. Neoplasia.

[66] Bi J., Tian Z. (2019). NK Cell Dysfunction and Checkpoint Immunotherapy. Front. Immunol..

[67] Dasgupta S., Bhattacharya-Chatterjee M., O’Malley B.W., Chatterjee S.K. (2005). Inhibition of NK Cell Activity through TGF-β1 by Down-Regulation of NKG2D in a Murine Model of Head and Neck Cancer. J. Immunol..

[68] Duan S., Guo W., Xu Z., He Y., Liang C., Mo Y., Wang Y., Xiong F., Guo C., Li Y. (2019). Natural killer group 2D receptor and its ligands in cancer immune escape. Mol. Cancer.

[69] Mistry A.R., O’Callaghan C.A. (2007). Regulation of ligands for the activating receptor NKG2D. Immunology.

[70] Harrison D., Phillips J.H., Lanier L.L. (1991). Involvement of a metalloprotease in spontaneous and phorbol ester-induced release of natural killer cell-associated Fc gamma RIII (CD16-II). J. Immunol..

[71] Liu Y., Cheng Y., Xu Y., Wang Z., Du X., Li C., Peng J., Gao L., Liang X., Ma C. (2017). Increased expression of programmed cell death protein 1 on NK cells inhibits NK-cell-mediated anti-tumor function and indicates poor prognosis in digestive cancers. Oncogene.

[72] Stojanovic A., Cerwenka A. (2011). Natural Killer Cells and Solid Tumors. J. Innate Immun..

[73] Parihar R., Rivas C.H., Huynh M., Omer B., Lapteva N., Metelitsa L.S., Gottschalk S.M., Rooney C.M. (2019). NK Cells Expressing a Chimeric Activating Receptor Eliminate MDSCs and Rescue Impaired CAR-T Cell Activity against Solid Tumors. Cancer Immunol. Res..

[74] Chang Y.-H., Connolly J., Shimasaki N., Mimura K., Kono K., Campana D. (2013). A Chimeric Receptor with NKG2D Specificity Enhances Natural Killer Cell Activation and Killing of Tumor Cells. Cancer Res..

[75] Xiao L., Cen D., Gan H., Sun Y., Huang N., Xiong H., Jin Q., Su L., Liu X., Wang K. (2019). Adoptive Transfer of NKG2D CAR mRNA-Engineered Natural Killer Cells in Colorectal Cancer Patients. Mol. Ther..

[76] Santana V.M., Barfield R.C. (2010). Anti-GD2 Antibody Therapy for GD2-Expressing Tumors. Curr. Cancer Drug Targets.

[77] Garnock-Jones K.P., Keating G.M., Scott L.J. (2010). Trastuzumab. Drugs.

[78] García-Foncillas J., Díaz-Rubio E. (2010). Progress in metastatic colorectal cancer: Growing role of cetuximab to optimize clinical outcome. Clin. Transl. Oncol..

[79] Winter M., Hancock B.W. (2009). Ten years of rituximab in NHL. Expert Opin. Drug Saf..

[80] Albertini M.R., Hank J.A., Sondel P.M. (2005). Native and genetically engineered anti-disialoganglioside monoclonal antibody treatment of melanoma. Cancer Chemother. Biol. Response Modif. Annu..

[81] Yang R.K., Sondel P.M. (2010). Anti-GD2 Strategy in the Treatment of Neuroblastoma. Drugs Futur..

[82] Parihar R., Nadella P., Lewis A., Jensen R., De Hoff C., Dierksheide J.E., VanBuskirk A.M., Magro C.M., Young N.C., Shapiro C.L. (2004). A Phase I Study of Interleukin 12 with Trastuzumab in Patients with Human Epidermal Growth Factor Receptor-2-Overexpressing Malignancies: Analysis of Sustained Interferon Production in a Subset of Patients. Clin. Cancer Res..

[83] Fleming G.F., Meropol N.J., Rosner G.L., Hollis D.R., Carson W.E., Caligiuri M., Mortimer J., Tkaczuk K., Parihar R., Schilsky R.L. (2002). A phase I trial of escalating doses of trastuzumab combined with daily subcutaneous interleukin 2: Report of cancer and leukemia group B 9661. Clin. Cancer Res..

[84] Snyder K.M., Hullsiek R., Mishra H.K., Mendez D.C., Li Y., Rogich A., Kaufman D.S., Wu J., Walcheck B. (2018). Expression of a Recombinant High Affinity IgG Fc Receptor by Engineered NK Cells as a Docking Platform for Therapeutic mAbs to Target Cancer Cells. Front. Immunol..

[85] Zhu H., Blum R.H., Bjordahl R., Gaidarova S., Rogers P., Lee T.T., Abujarour R., Bonello G.B., Wu J., Tsai P.-F. (2020). Pluripotent stem cell–derived NK cells with high-affinity noncleavable CD16a mediate improved antitumor activity. Blood.

[86] Pomeroy E.J., Hunzeker J.T., Kluesner M.G., Lahr W.S., Smeester B.A., Crosby M.R., Lonetree C.-L., Yamamoto K., Bendzick L., Miller J.S. (2020). A Genetically Engineered Primary Human Natural Killer Cell Platform for Cancer Immunotherapy. Mol. Ther..

[87] Savan R., Chan T., Young H.A. (2010). Lentiviral gene transduction in human and mouse NK cell lines. Adv. Struct. Saf. Stud..

[88] Matosevic S. (2018). Viral and Nonviral Engineering of Natural Killer Cells as Emerging Adoptive Cancer Immunotherapies. J. Immunol. Res..

[89] Afolabi L.O., Adeshakin A.O., Sani M.M., Bi J., Wan X. (2019). Genetic reprogramming for NK cell cancer immunotherapy with CRISPR/Cas9. Immunology.

[90] Carlsten M., Childs R.W. (2015). Genetic Manipulation of NK Cells for Cancer Immunotherapy: Techniques and Clinical Implications. Front. Immunol..

[91] Khan M., Arooj S., Wang H. (2020). NK Cell-Based Immune Checkpoint Inhibition. Front. Immunol..

[92] André P., Denis C., Soulas C., Bourbon-Caillet C., Lopez J., Arnoux T., Bléry M., Bonnafous C., Gauthier L., Morel A. (2018). Anti-NKG2A mAb Is a Checkpoint Inhibitor that Promotes Anti-tumor Immunity by Unleashing Both T and NK Cells. Cell.

[93] Moretta A., Bottino C., Vitale M., Pende D., Biassoni R., Mingari M.C., Moretta L. (1996). Receptors for HLA Class-I molecules in human natural killer cells. Annu. Rev. Immunol..

[94] Moretta A., Bottino C., Pende D., Tripodi G., Tambussi G., Viale O., Orengo A., Barbaresi M., Merli A., Ciccone E. (1990). Identification of four subsets of human CD3-CD16+ natural killer (NK) cells by the expression of clonally distributed functional surface molecules: Correlation between subset assignment of NK clones and ability to mediate specific alloantigen recognition. J. Exp. Med..

[95] Sola C., Chanuc F., Thielens A., Fuseri N., Morel Y., Bléry M., André P., Vivier E., Graziano R., Romagne F. (2013). Anti-tumoral efficacy of therapeutic human anti-KIR antibody (Lirilumab/BMS-986015/IPH2102) in a preclinical xenograft tumor model. J. Immunother. Cancer.

[96] Vey N., Karlin L., Sadot-Lebouvier S., Broussais F., Berton-Rigaud D., Rey J., Charbonnier A., Marie D., André P., Paturel C. (2018). A phase 1 study of lirilumab (antibody against killer immunoglobulin-like receptor antibody KIR2D; IPH2102) in patients with solid tumors and hematologic malignancies. Oncotarget.

[97] Zhang C.-X., Huang D.-J., Baloche V., Zhang L., Xu J.-X., Li B.-W., Zhao X.-R., He J., Mai H.-Q., Chen Q.-Y. (2020). Galectin-9 promotes a suppressive microenvironment in human cancer by enhancing STING degradation. Oncogenesis.

[98] Ndhlovu L.C., Lopez-Vergès S., Barbour J.D., Jones R.B., Jha A.R., Long B.R., Schoeffler E.C., Fujita T., Nixon D.F., Lanier L.L. (2012). Tim-3 marks human natural killer cell maturation and suppresses cell-mediated cytotoxicity. Blood.

[99] Farkas A.M., Audenet F., Anastos H., Galsky M., Sfakianos J., Bhardwaj N. (2018). Tim-3 and TIGIT mark Natural Killer cells susceptible to effector dysfunction in human bladder cancer. J. Immunol..

[100] Da Silva I.P., Gallois A., Jimenez-Baranda S., Khan S., Anderson A.C., Kuchroo V.K., Osman I., Bhardwaj N. (2014). Reversal of NK-Cell Exhaustion in Advanced Melanoma by Tim-3 Blockade. Cancer Immunol. Res..

[101] Xu L., Huang Y., Tan L., Yu W., Chen D., Lu C., He J., Wu G., Liu X., Zhang Y. (2015). Increased Tim-3 expression in peripheral NK cells predicts a poorer prognosis and Tim-3 blockade improves NK cell-mediated cytotoxicity in human lung adenocarcinoma. Int. Immunopharmacol..

[102] He X., Xu C. (2020). Immune checkpoint signaling and cancer immunotherapy. Cell Res..

[103] Yin X., Liu T., Wang Z., Ma M., Lei J., Zhang Z., Fu S., Fu Y., Hu Q., Ding H. (2018). Expression of the Inhibitory Receptor TIGIT Is Up-Regulated Specifically on NK Cells With CD226 Activating Receptor From HIV-Infected Individuals. Front. Immunol..

[104] Zhang Q., Bi J., Zheng X., Chen Y., Wang H., Wu W., Wang Z., Wu Q., Peng H., Wei H. (2018). Blockade of the checkpoint receptor TIGIT prevents NK cell exhaustion and elicits potent anti-tumor immunity. Nat. Immunol..

[105] Maas R.J., Evert J.S.H.-V., Van Der Meer J.M., Mekers V., Rezaeifard S., Korman A.J., De Jonge P.K., Cany J., Woestenenk R., Schaap N.P. (2020). TIGIT blockade enhances functionality of peritoneal NK cells with altered expression of DNAM-1/TIGIT/CD96 checkpoint molecules in ovarian cancer. OncoImmunology.

[106] Judge S.J., Darrow M.A., Thorpe S.W., Gingrich A.A., O’Donnell E.F., Bellini A.R., Sturgill I.R., Vick L.V., Dunai C., Stoffel K.M. (2020). Analysis of tumor-infiltrating NK and T cells highlights IL-15 stimulation and TIGIT blockade as a combination immunotherapy strategy for soft tissue sarcomas. J. Immunother. Cancer.

[107] Gleason M.K., Ross J.A., Warlick E.D., Lund T.C., Verneris M.R., Wiernik A., Spellman S., Haagenson M.D., Lenvik A.J., Litzow M.R. (2014). CD16xCD33 bispecific killer cell engager (BiKE) activates NK cells against primary MDS and MDSC CD33+ targets. Blood.

[108] Sarhan D., Brandt L., Felices M., Guldevall K., Lenvik T., Hinderlie P., Curtsinger J., Warlick E., Spellman S.R., Blazar B.R. (2018). 161533 TriKE stimulates NK-cell function to overcome myeloid-derived suppressor cells in MDS. Blood Adv..

[109] Vallera D.A., Felices M., McElmurry R., McCullar V., Zhou X., Schmohl J.U., Zhang B., Lenvik A.J.L., Panoskaltsis-Mortari A., Verneris M.R. (2016). IL15 Trispecific Killer Engagers (TriKE) Make Natural Killer Cells Specific to CD33+ Targets While Also Inducing Persistence, In Vivo Expansion, and Enhanced Function. Clin. Cancer Res..

[110] Vallera D.A., Ferrone S., Kodal B., Hinderlie P., Bendzick L., Ettestad B., Hallstrom C., Zorko N.A., Rao A., Fujioka N. (2020). NK-Cell-Mediated Targeting of Various Solid Tumors Using a B7-H3 Tri-Specific Killer Engager In Vitro and In Vivo. Cancers.

[111] Schmohl J.U., Felices M., Taras E., Miller J.S., Vallera D.A. (2016). Enhanced ADCC and NK Cell Activation of an Anticarcinoma Bispecific Antibody by Genetic Insertion of a Modified IL-15 Cross-linker. Mol. Ther..

[112] Hu W., Wang G., Huang D.-S., Sui M., Xu Y. (2019). Cancer Immunotherapy Based on Natural Killer Cells: Current Progress and New Opportunities. Front. Immunol..

[113] Minetto P., Guolo F., Pesce S., Greppi M., Obino V., Ferretti E., Sivori S., Genova C., Lemoli R.M., Marcenaro E. (2019). Harnessing NK Cells for Cancer Treatment. Front. Immunol..

[114] Lapteva N., Szmania S.M., Van Rhee F., Rooney C.M. (2013). Clinical Grade Purification and Expansion of Natural Killer Cells. Crit. Rev. Oncog..

[115] Zhu H., Kaufman D.S. (2019). An Improved Method to Produce Clinical-Scale Natural Killer Cells from Human Pluripotent Stem Cells. Methods Mol. Biol..

[116] Vasu S., Berg M., Davidsonmoncada J.K., Tian X., Cullis H., Childs R.W. (2015). A novel method to expand large numbers of CD56+ natural killer cells from a minute fraction of selectively accessed cryopreserved cord blood for immunotherapy after transplantation. Cytotherapy.

[117] Ojo E.O., Sharma A.A., Liu R., Moreton S., Checkley-Luttge M.-A., Gupta K., Lee G., Lee D.A., Otegbeye F., Sekaly R.-P. (2019). Membrane bound IL-21 based NK cell feeder cells drive robust expansion and metabolic activation of NK cells. Sci. Rep..

[118] Williams B.A., Law A.D., Routy B., Denhollander N., Gupta V., Wang X.-H., Chaboureau A., Viswanathan S., Keating A. (2017). A phase I trial of NK-92 cells for refractory hematological malignancies relapsing after autologous hematopoietic cell transplantation shows safety and evidence of efficacy. Oncotarget.

[119] Matsuo Y., Drexler H.G. (2003). Immunoprofiling of cell lines derived from natural killer-cell and natural killer-like T-cell leukemia–lymphoma. Leuk. Res..

[120] Jochems C., Hodge J.W., Fantini M., Fujii R., Ii Y.M.M., Greiner J.W., Padget M.R., Tritsch S.R., Tsang K.Y., Campbell K.S. (2016). An NK cell line (haNK) expressing high levels of granzyme and engineered to express the high affinity CD16 allele. Oncotarget.

[121] Zhang J., Zheng H., Diao Y. (2019). Natural Killer Cells and Current Applications of Chimeric Antigen Receptor-Modified NK-92 Cells in Tumor Immunotherapy. Int. J. Mol. Sci..

[122] Borst L., Van Der Burg S.H., Van Hall T. (2020). The NKG2A–HLA-E Axis as a Novel Checkpoint in the Tumor Microenvironment. Clin. Cancer Res..

[123] Noman M.Z., DeSantis G., Janji B., Hasmim M., Karray S., Dessen P., Bronte V., Chouaib S. (2014). PD-L1 is a novel direct target of HIF-1α, and its blockade under hypoxia enhanced MDSC-mediated T cell activation. J. Exp. Med..

[124] Hu Y., Tian Z.-G., Zhang C. (2017). Chimeric antigen receptor (CAR)-transduced natural killer cells in tumor immunotherapy. Acta Pharm. Ther. Sin..

[125] Janelle V. (2019). The Bumpy Road to CAR Activation. Mol. Ther..

[126] Poli A., Michel T., Thérésine M., Andrès E., Hentges F., Zimmer J. (2008). CD56brightnatural killer (NK) cells: An important NK cell subset. Immunology.

[127] Wagner J.A., Rosario M., Romee R., Berrien-Elliott M.M., Schneider S.E., Leong J.W., Sullivan R.P., Jewell B.A., Becker-Hapak M., Schappe T. (2017). CD56bright NK cells exhibit potent antitumor responses following IL-15 priming. J. Clin. Investig..

[128] Gunesch J.T., Dixon A.L., Ebrahim T.A., Berrien-Elliott M.M., Tatineni S., Kumar T., Hegewisch-Solloa E., Fehniger T.A., Mace E.M. (2020). CD56 regulates human NK cell cytotoxicity through Pyk2. eLife.

[129] De Jonge K., Ebering A., Nassiri S., Hajjami H.M.-E., Ouertatani-Sakouhi H., Baumgaertner P., Speiser D.E. (2019). Circulating CD56bright NK cells inversely correlate with survival of melanoma patients. Sci. Rep..

[130] Vahedi F., Nham T., Poznanski S.M., Chew M.V., Shenouda M.M., Lee D., Ashkar A. (2017). Ex Vivo Expanded Human NK Cells Survive and Proliferate in Humanized Mice with Autologous Human Immune Cells. Sci. Rep..

[131] Hoogi S., Eisenberg V., Mayer S., Shamul A., Barliya T., Cohen C.J. (2019). A TIGIT-based chimeric co-stimulatory switch receptor improves T-cell anti-tumor function. J. Immunother. Cancer.

[132] Liu X., Ranganathan R., Jiang S., Fang C., Sun J., Kim S., Newick K., Lo A., June C.H., Zhao Y. (2016). A Chimeric Switch-Receptor Targeting PD1 Augments the Efficacy of Second-Generation CAR T Cells in Advanced Solid Tumors. Cancer Res..

[133] Liu H., Wang S., Xin J., Wang J., Yao C., Zhang Z. (2019). Role of NKG2D and its ligands in cancer immunotherapy. Am. J. Cancer Res..

[134] Sambi M., Bagheri L., Szewczuk M.R. (2019). Current Challenges in Cancer Immunotherapy: Multimodal Approaches to Improve Efficacy and Patient Response Rates. J. Oncol..

[135] Weber R., Fleming V., Hu X., Nagibin V., Groth C., Altevogt P., Utikal J., Umansky V. (2018). Myeloid-Derived Suppressor Cells Hinder the Anti-Cancer Activity of Immune Checkpoint Inhibitors. Front. Immunol..

[136] Trombetta A.C., Soldano S., Contini P., Tomatis V., Ruaro B., Paolino S., Brizzolara R., Montagna P., Sulli A., Pizzorni C. (2018). A circulating cell population showing both M1 and M2 monocyte/macrophage surface markers characterizes systemic sclerosis patients with lung involvement. Respir. Res..

[137] Kararoudi M.N., Dolatshad H., Trikha P., Hussain S.-R.A., Elmas E., Foltz J.A., Moseman J.E., Thakkar A., Nakkula R.J., Lamb M. (2018). Generation of Knock-out Primary and Expanded Human NK Cells Using Cas9 Ribonucleoproteins. J. Vis. Exp..

[138] Kitada T., DiAndreth B., Teague B., Weisst R. (2018). Programming gene and engineered-cell therapies with synthetic biology. Science.

[139] Kakarla S., Chow K.K.H., Mata M., Shaffer D.R., Song X.-T., Wu M.-F., Liu H., Wang L.L., Rowley D.R., Pfizenmaier K. (2013). Antitumor Effects of Chimeric Receptor Engineered Human T Cells Directed to Tumor Stroma. Mol. Ther..

[140] Tran E., Chinnasamy D., Yu Z., Morgan R.A., Lee C.-C.R., Restifo N.P., Rosenberg S.A. (2013). Immune targeting of fibroblast activation protein triggers recognition of multipotent bone marrow stromal cells and cachexia. J. Exp. Med..

[141] Schuberth P.C., Hagedorn C., Jensen S.M., Gulati P., Broek M.V.D., Mischo A., Soltermann A., Jüngel A., Belaunzaran O.M., Stahel R.A. (2013). Treatment of malignant pleural mesothelioma by fibroblast activation protein-specific re-directed T cells. J. Transl. Med..

[142] Brodin P., Lakshmikanth T., Johansson S., Kärre K., Höglund P. (2009). The strength of inhibitory input during education quantitatively tunes the functional responsiveness of individual natural killer cells. Blood.

[143] Carisey A.F., Mace E.M., Saeed M.B., Davis D.M., Orange J.S. (2018). Nanoscale Dynamism of Actin Enables Secretory Function in Cytolytic Cells. Curr. Biol..

[144] Jenkins M.R., Tsun A., Stinchcombe J.C., Griffiths G.M. (2009). The Strength of T Cell Receptor Signal Controls the Polarization of Cytotoxic Machinery to the Immunological Synapse. Immunology.

[145] Beal A.M., Anikeeva N., Varma R., Cameron T.O., Vasiliver-Shamis G., Norris P.J., Dustin M.L., Sykulev Y. (2009). Kinetics of Early T Cell Receptor Signaling Regulate the Pathway of Lytic Granule Delivery to the Secretory Domain. Immunology.

[146] Rafiq S., Yeku O.O., Jackson H.J., Purdon T.J., Van Leeuwen D.G., Drakes D.J., Song M., Miele M.M., Li Z., Wang P. (2018). Targeted delivery of a PD-1-blocking scFv by CAR-T cells enhances anti-tumor efficacy in vivo. Nat. Biotechnol..

[147] Suarez E.R., Chang D.-K., Sun J., Sui J., Freeman G.J., Signoretti S., Zhu Q., Marasco W.A. (2016). Chimeric antigen receptor T cells secreting anti-PD-L1 antibodies more effectively regress renal cell carcinoma in a humanized mouse model. Oncotarget.

[148] Jin L., Tao H., Karachi A., Long Y., Hou A.Y., Na M., Dyson K.A., Grippin A.J., Deleyrolle L.P., Zhang W. (2019). CXCR1- or CXCR2-modified CAR T cells co-opt IL-8 for maximal antitumor efficacy in solid tumors. Nat. Commun..

[149] Ng Y.Y., Tay J.C., Wang S. (2020). CXCR1 Expression to Improve Anti-Cancer Efficacy of Intravenously Injected CAR-NK Cells in Mice with Peritoneal Xenografts. Mol. Ther. Oncolytics.

[150] Costa E.C., Moreira A.F., De Melo-Diogo D., Gaspar V.M., Carvalho M.P., Correia I. (2016). 3D tumor spheroids: An overview on the tools and techniques used for their analysis. Biotechnol. Adv..

[151] Wang M., Yao L., Cheng M., Cai D., Martinek J., Pan C., Shi W., Ma A., White R.W.D.V., Airhart S. (2018). Humanized mice in studying efficacy and mechanisms of PD-1-targeted cancer immunotherapy. FASEB J..

[152] Zhao Y., Shuen T.W.H., Toh T.B., Chan X.Y., Liu M., Tan S.Y., Fan Y., Yang H., Lyer S.G., Bonney G.K. (2018). Development of a new patient-derived xenograft humanised mouse model to study human-specific tumour microenvironment and immunotherapy. Gut.

[153] Lin S., Huang G., Cheng L., Li Z., Xiao Y., Deng Q., Jiang Y., Li B., Lin S., Wang S. (2018). Establishment of peripheral blood mononuclear cell-derived humanized lung cancer mouse models for studying efficacy of PD-L1/PD-1 targeted immunotherapy. mAbs.

[154] Martins F., Sofiya L., Sykiotis G.P., Lamine F., Maillard M., Fraga M., Shabafrouz K., Ribi C., Cairoli A., Guex-Crosier Y. (2019). Adverse effects of immune-checkpoint inhibitors: Epidemiology, management and surveillance. Nat. Rev. Clin. Oncol..

[155] Oliva A., Kinter A.L., Vaccarezza M., Rubbert A., Catanzaro A., Moir S., Monaco J., Ehler L., Mizell S., Jackson R. (1998). Natural killer cells from human immunodeficiency virus (HIV)-infected individuals are an important source of CC-chemokines and suppress HIV-1 entry and replication in vitro. J. Clin. Investig..

[156] A Fehniger T., Shah M.H., Turner M.J., VanDeusen J.B., Whitman S.P., A Cooper M., Suzuki K., Wechser M., Goodsaid F., Caligiuri M.A. (1999). Differential cytokine and chemokine gene expression by human NK cells following activation with IL-18 or IL-15 in combination with IL-12: Implications for the innate immune response. J. Immunol..

[157] Roda J.M., Parihar R., Magro C., Nuovo G.J., Tridandapani S., Carson W.E. (2006). Natural Killer Cells Produce T Cell–Recruiting Chemokines in Response to Antibody-Coated Tumor Cells. Cancer Res..

[158] Wang J., Matosevic S. (2018). Adenosinergic signaling as a target for natural killer cell immunotherapy. J. Mol. Med..

[159] Akhiani A.A., Hallner A., Kiffin R., Aydin E., Werlenius O., Aurelius J., Martner A., Thorén F.B., Hellstrand K. (2020). Idelalisib Rescues Natural Killer Cells from Monocyte-Induced Immunosuppression by Inhibiting NOX2-Derived Reactive Oxygen Species. Cancer Immunol. Res..

[160] Cekic C., Day Y.-J., Sag D., Linden J. (2014). Myeloid Expression of Adenosine A2A Receptor Suppresses T and NK Cell Responses in the Solid Tumor Microenvironment. Cancer Res..

[161] Ghalamfarsa G., Kazemi M.H., Mohseni S.R., Masjedi A., Hojjat-Farsangi M., Azizi G., Yousefi M., Jadidi-Niaragh F. (2018). CD73 as a potential opportunity for cancer immunotherapy. Expert Opin. Ther. Targets.

[162] Neo S.Y., Yang Y., Record J., Ma R., Chen X., Chen Z., Tobin N.P., Blake E., Seitz C., Thomas R. (2020). CD73 immune checkpoint defines regulatory NK cells within the tumor microenvironment. J. Clin. Investig..

[163] Michelet X., Dyck L., Hogan A., Loftus R.M., Duquette D., Wei K., Beyaz S., Tavakkoli A., Foley C., Donnelly R. (2018). Metabolic reprogramming of natural killer cells in obesity limits antitumor responses. Nat. Immunol..

[164] Tobin L.M., Mavinkurve M., Carolan E., Kinlen D., O’Brien E.C., Little M.A., Finlay D.K., Cody D., Hogan A.E., O’Shea D. (2017). NK cells in childhood obesity are activated, metabolically stressed, and functionally deficient. JCI Insight.

[165] Harmon C., Robinson M.W., Hand F., AlMuaili D., Mentor K., Houlihan D.D., Hoti E., Lynch L., Geoghegan J., O’Farrelly C. (2018). Lactate-Mediated Acidification of Tumor Microenvironment Induces Apoptosis of Liver-Resident NK Cells in Colorectal Liver Metastasis. Cancer Immunol. Res..

[166] Cassim S., Pouyssegur J. (2019). Tumor Microenvironment: A Metabolic Player that Shapes the Immune Response. Int. J. Mol. Sci..

[167] Pająk B., Siwiak E., Sołtyka M., Priebe A., Zieliński R., Fokt I., Ziemniak M., Jaśkiewicz A., Borowski R., Domoradzki T. (2019). 2-Deoxy-d-Glucose and Its Analogs: From Diagnostic to Therapeutic Agents. Int. J. Mol. Sci..

[168] Xu X., Meng Y., Li L., Xu P., Wang J., Li Z., Bian J. (2019). Overview of the Development of Glutaminase Inhibitors: Achievements and Future Directions. J. Med. Chem..

[169] Dhankhar R., Gupta V., Kumar S., Kapoor R.K., Gulati P. Microbial Enzymes for Deprivation of Amino Acid Metabolism in Malignant Cells: Biological Strategy for Cancer Treatment. https://pubmed.ncbi.nlm.nih.gov/32037468/.

[170] Mah A.Y., Rashidi A., Keppel M.P., Saucier N., Moore E.K., Alinger J.B., Tripathy S.K., Agarwal S.K., Jeng E.K., Wong H.C. (2017). Glycolytic requirement for NK cell cytotoxicity and cytomegalovirus control. JCI Insight.

[171] Ando M., Sugimoto K., Itoh Y., Sasaki M., Mukai K., Ando J., Egashira M., Schuster S.M., Oshimi K. (2005). Selective apoptosis of natural killer-cell tumours by l-asparaginase. Br. J. Haematol..

